# Ontology-based cross-species integration and analysis of Saccharomyces cerevisiae phenotypes

**DOI:** 10.1186/2041-1480-3-S2-S6

**Published:** 2012-09-21

**Authors:** Georgios V Gkoutos, Robert Hoehndorf

**Affiliations:** 1 Department of Genetics, University of Cambridge, Downing Street, Cambridge, Cambridge CB2 3EH, UK; 2 Department of Computer Science, University of Aberystwyth, Old College, King Street, SY23 2AX, UK

## Abstract

Ontologies are widely used in the biomedical community for annotation and integration of databases. Formal definitions can relate classes from different ontologies and thereby integrate data across different levels of granularity, domains and species. We have applied this methodology to the Ascomycete Phenotype Ontology (APO), enabling the reuse of various orthogonal ontologies and we have converted the phenotype associated data found in the SGD following our proposed patterns. We have integrated the resulting data in the cross-species phenotype network *PhenomeNET*, and we make both the cross-species integration of yeast phenotypes and a similarity-based comparison of yeast phenotypes across species available in the PhenomeBrowser. Furthermore, we utilize our definitions and the yeast phenotype annotations to suggest novel functional annotations of gene products in yeast.

## Background

Yeast phenotypes have been proven useful for investigating and revealing various aspects of cellular physiology and mechanisms. The study of these phenotypes has direct implications for understanding mammalian physiology in the context of pharmacodynamics and pharmacokinetics studies, in understanding signalling and regulatory networks, in studies that focus on the identification of response regulators, activators and inhibitors, and in chemical genetics [1-4]. It is therefore essential that efficient ways are set in place to collect and analyse yeast phenotype data as well as compare them with other organism phenotypes held in a variety of resources.

Over the last years, a plethora of phenotype ontologies has been proposed [5-11]. These ontologies are developed by a variety of biomedical communities and aim to support the annotation of phenotypic observations derived either from the literature or from experimental studies, including large scale phenotype studies [12,13]. To unify the species-specific efforts in representing phenotypes, to enable the integration of phenotype information across species, and to enhance the formally represented genotype-to-phenotype knowledge, a species and domain independent method for decomposing phenotypes was proposed based on the Phenotype And Trait Ontology [14]. This method has been successfully applied both for the direct annotation of species-specific phenotypes and for defining classes in species-specific phenotype ontologies to enable cross-species phenotype integration [15-18].

The *Saccharomyces *Genome Database (SGD) [19] collects and curates yeast-related phenotype data using the yeast-specific Ascomycete Phenotype Ontology (APO) [20]. Here, we report our efforts to apply the EQ-based method to the APO and enable the reuse of biomedical reference ontologies to describe yeast-related phenotype information as well as integrate it with other species. We apply the results of our analysis to the cross-species phenotype network PhenomeNET [21] and make both the cross-species integration of yeast phenotypes and a similarity-based comparison of yeast phenotypes across species available in the PhenomeBrowser [22].

## Materials and methods

### Saccharomyces Genome Database

The *Saccharomyces *Genome Database (SGD) is a freely available collection of genetic and molecular information about *Saccharomyces cerevisiae*. The SGD contains, amongst others, sequence information for yeast genes and proteins as well as tools for their analyses and comparison, descriptions of their biological roles and molecular functions, the subcellular location at which proteins are active, literature information and links to external resources [19].

In particular, SGD contains information about phenotypes that arise from curation of either the published scientific literature of traditional bench experiments or from the results of a number of large-scale studies [20]. Such information can be useful for revealing new molecular functional information of genes and SGD curators currently focus on its integration with the available genetic information [19]. The phenotype information recorded includes developmental, metabolism and growth related, processual and morphological manifestations at the cellular level [20].

### PATO

The Phenotype And Trait Ontology (PATO) was envisaged and designed to provide a platform for allowing the integration of quantitative and qualitative phenotype related information across different levels of granularity of domain knowledge granularity and species [14]. PATO provides an ontology of phenotype related qualities that compromise the basic entities that we can perceive and/or measure such as colors, sizes, masses, lengths etc. Qualities inhere to entities: every entity comes with certain qualities that exist as long as the entity exists. PATO allows for the description of affected entities by combining various ontologies that describe the entities that have been affected, such as the various anatomical ontologies, GO [23], the Cell Type Ontology [24] or the Sequence Ontology (SO) [25], with the various qualities it provides for defining how these entities were affected. PATO can be used for annotation either directly in a so called post-composed (post-coordinated) manner or for providing logical definitions (equivalence axioms) to ontologies containing a set of precomposed (pre-coordinated) phenotype terms. For instance, to describe a developmental delay phenotype of yeast cell, we can combine the PATO term *delayed *(PATO:0000502) with the GO term *cellular development process *(GO:0048869), whilst if such a term existed in a precomposed ontology it could be used to provide an equivalence statement between that class and the above PATO-based description.

### Annotating phenotypes using the Ascomycete Phenotype Ontology

**Figure 1:**
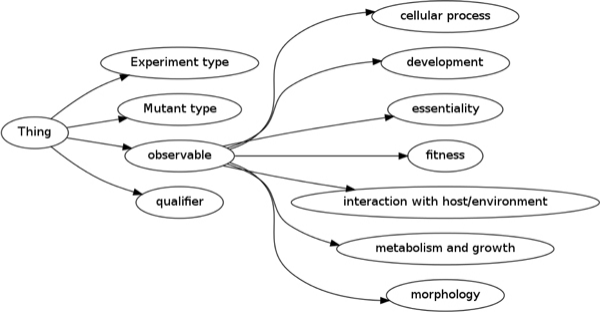
**Top-level of the Ascomycete Phenotype Ontology**.

The curation of yeast phenotype information is based on a combination of multiple controlled vocabularies which are available from the OBO Foundry ontology repository [26]. One of these vocabularies is the Ascomycete Phenotype Ontology (APO) that, as of 30/06/2011, contains 269 terms organised in four hierarchies [20]. Sub-classes of *Experiment type *provide a classification of genetic interactions and types of experiments (assays) performed on yeast. The class *Mutant type *has sub-classes that provide a classification of types of mutations in yeast that may cause a specific phenotype. Finally, the *observable *and *qualifier *classes are used to record the actual phenotypic observation [20]. The top-level classes of the APO are shown in Figure 1.

According to APO, the *observable *class corresponds to the feature or the trait of a phenotype. For example, traits that can be sub-classes of the *observable *class include the *shape *or *size *of a cell or the *rate *of a growth. These sub-classes are distinguished based on the entity that is affected in a phenotype manifestation and based on the *trait *that is affected. For example, classification based on the entity yields *cellular process*, *cell metabolism *and *cellular growth*, while the classification based on traits results in sub-classes such as *cell morphology*. The APO's *qualifier *class, on the other hand, provides a set of possible comparative values for these traits. For example, *increased, arrested *and *abnormal *are included as sub-classes of APO's *qualifier *class. In order to annotate a phenotype corresponding to the observation of *abnormal cell shape*, the APO class *cell shape *(APO:0000051) (a sub-class of *observable*) is combined with the APO class abnormal (APO:0000002) (a sub-class of *qualifier*). APO terms can further be used in conjunction with further ontologies, in particular the Chemical Entities of Biological Interest (ChEBI) ontology [27] to extent their ability to describe phenotypes.

## Results

To formally decompose APO's phenotype classes based on the EQ method and enable the integration of yeast phenotype annotations with phenotype annotations from other species, we have used the PATO [14] and the Gene Ontology (GO) [23] as well as ChEBI [27]. We apply different definition patterns for the different sub-classes of APO's observable.

### Morphological traits

APO morphological characteristics are applicable to the morphology of either cellular or sub-cellular structures. We have used the class *Morphology *(PATO:0000051) and its subclasses, and we link them to the appropriate anatomical localisation provided by GO's cellular component branch. For example, to define the APO term *Cell wall morphology *(APO:0000053), the GO cellular anatomical term *Cell wall *(GO:0005618) is linked to the *Morphology *(PATO:0000051) term from the PATO ontology.

We implement this EQ-based definition in the OBO Flat file Format [28] following the syntactic patterns associated with EQ [18]. In the OBO Flat file Format, the definition can be expressed as follows:

[Term]

id: APO:0000053 ! cell wall morphology

intersection_of: PATO:0000051 ! morphology

intersection_of: inheres_in GO:0005618

Formally, we use the conversion approach used in the PhenomeBLAST software [22] to represent this syntactic description of a phenotype in OWL. PhenomeBLAST applies a simplified form of the phene-patterns [29], and the *Cell wall morphology *phenotype would be represented as a phenotype of entities that have a cell wall as part in which a quality of the type *Morphology *inheres:

APO:0000053 EquivalentTo: phenotype-of some

   (has-part some (GO:0005618 and

   has-quality some PATO:0000051))

In some cases, the APO terms are related to temporal stages, i.e., the phenotypes are observed only while the yeast cell is in a certain stage. For example, stages of the cell cycle are used in classes such as *Critical cell size at G2/M (cryptic G2/M cell size checkpoint) *(APO:0000142). To define a class involving reference to a temporal stage, we use the **during **relation and a class from the GO. In the OBO Flat file Format, the class *Critical cell size at G2/M (cryptic G2/M cell size checkpoint) *is defined as follows:

[Term]

id: APO:0000142

intersection_of: PATO:0000117 ! size

intersection_of: inheres_in GO:0005623

intersection_of: during GO:0031576

Formally, this phenotype is translated into the OWL definition:

APO:0000142 EquivalentTo: phenotype-of some

   (has-part some (GO:0005623 and

   has-quality some PATO:0000117 and

   during some GO:0031576))

### Developmental, metabolic and physiological phenotypes

The APO contains the classes *Cellular process*, *Development*, *Metabolism and growth *as well as *Interaction with host/environment*. We assume that each of these classes represents a phenotype that is based on a process. In particular, we use GO's classification of processes to define the APO class *Cellular process *(APO:0000066) as a phenotype of a *Cellular process *(GO:0009987), *Development *(APO:0000023) as a phenotype of a *Cellular developmental process *(GO:0048869) and *Metabolism and growth *(APO:0000094) as a phenotype of either *Cellular metabolic process *(GO:0044237) or *Cellular growth *(GO:0016049). To obtain additional inferences based on the parthood relations in the GO, we use definition patterns that include the **part-of **relation, which we assume to be reflexive and transitive. For example, we formally define *Cellular process *as:

APO:0000066 EquivalentTo: phenotype-of some

   (has-part some (part-of some

     GO:0009987 and has-quality some

     PATO:0000001))

This definition pattern uses the **has-part **relation to relate an organism (the range of **phenotype-of**) to a process. We do not use the **participates-in **relation for this purpose, since explicitly distinguishing between processes and material objects will currently lead to contradictions in phenotype ontologies and the GO [30]. In the future, we intend to explicitly incorporate more expressive phenotype definition patterns that enable interoperability between ontologies of both anatomy and physiology [29].

To define APO classes that describe phenotypes associated with biological processes or molecular functions, we linked the appropriate GO classes with terms from PATO. The classification of biological processes or molecular functions in the GO provide the entity affected by a phenotype while PATO characterizes *how *these entities are affected.

As a consequence of defining the sub-classes of *observable *in APO based on the GO using the **part-of **relation, we can infer a new and updated taxonomic structure of APO in which *Development *and *Metabolism and growth *are sub-classes of *Cellular process*. This inference is obtained through inference over GO's classification of processes and the definition patterns we provide.

### Dispositional phenotypes

A common kind of phenotypes in yeast include dispositions to interact with other substances in a particular way. For example, the APO class *Metal resistant *(APO:0000090) is used to describe yeast's disposition to interact with metal.

In the EQ-based decomposition of the class *Metal resistant*, we use GO's process class *Response to metal ion *(GO:0010038) and combine it with the PATO class *Sensitivity of a process *(PATO:0001457):

[Term]

id: APO:0000090

intersection_of: PATO:0001457

intersection_of: inheres_in GO:0010038

Similar to processual phenotypes, we do not yet use the **has-disposition **or **has-function **relation in formalizing this phenotype because formally distinguishing between functions and processes will lead to a large number of unsatisfiable class in phenotype ontologies and the GO. Consequently, we formally define *Metal resistant *as:

APO:0000090 EquivalentTo: phenotype-of some

     (has-part some (GO:0010038 and

     has-quality some PATO:0001457))

In the future, we intend to formalize dispositional phenotypes using the **has-disposition **or **has-function **relation.

### Interoperability with chemistry ontology

Relational classes from the PATO ontology can also be used to characterize qualities of more than one entity. We use the **towards **relation to specify the second argument of a relational quality. For example, we define the APO term *Resistance to chemicals *(APO:0000087) by linking the class *Chemical compound *(CHEBI:37577) to the PATO class *Sensitivity of a process *(PATO:0001457) and the process class *Response to chemical stimulus *(GO:0042221):

[Term]

id: APO:0000087

intersection_of: PATO:0001457

intersection_of: inheres_in GO:0042221

intersection_of: towards CHEBI:37577

Formally, we express this statement as

APO:0000087 EquivalentTo: phenotype-of some

     (GO:0042221 and

     has-quality some (PATO:0001457 and

      towards some CHEBI:37577))

### Phenotypic qualifiers

To relate APO's qualifier-classes to the PATO ontology, we created a statement of equivalency between PATO's qualifier classes and APO's qualifier classes. For example, for the APO term *arrested *(APO:0000250), we created an equivalent-class statement to the PATO term *arrested *(PATO:0000297). Since PATO formally distinguishes between qualities that inhere in objects and qualities that inhere in processes such statements also allowed for reasoners to automatically check the consistency of the combination of qualifiers with anatomical or processual terms created by curators for annotation purposes.

### Formalizing yeast phenotype annotations

The SGD makes phenotype annotations for specific genotypes and genetic interactions available. These annotations consist of a genotype identifier (such as S000029075) and either a pair or a triple of classes which describe the phenotype that is associated with the genotype. If the phenotype annotation consists of a pair of classes, a class from the APO's *observable *branch is combined with a class from the APO's *qualifier *branch. For example, the genotype S000029075, a conditional mutation of the *CDC29 *gene, has three phenotype annotations in the SGD:

• heat sensitivity (APO:0000147): increased (APO:0000004)

• budding (APO:0000024): absent (APO:0000005)

• cell cycle progression (APO:0000253): arrested (APO:0000250)

To formalize these phenotypes, we first identify the entity and the quality that is affected in a phenotype. For example, *Heat sensitivity *(APO:0000147) is defined as a phenotype of a *Response to heat *(GO:0009408) process and is based on the PATO quality *Sensitivity of a process *(PATO:0001457). Based on this information, we create an OWL class expression. Since the qualifier that is applied to *Heat sensitivity *(APO:0000147) is *Increased *(APO:0000004) and the quality *Sensitivity of a process *(PATO:0001457), we construct an anonymous *Increased sensitivity of a process *class using the **increased-in-magnitude-relative-to **(similarly to PATO's definition of the *Increased sensitivity of a process *class) (PATO:0001551)and formalize *Heat sensitivity: increased *as:

phenotype-of some (has-part some

 GO:0009408 and has-quality some

 (PATO:0001457 and

 increased-in-magnitude-relative-to some

 normal))

Based on this information, the phenotype description will be inferred to be a sub-class of APO's *Heat sensitivity*, it will inter-operate with phenotypes that are based on PATO's *Increased sensitivity of a process class *(because they share the same definition) and through inference over the GO we can obtain basic interoperability across multiple species' phenotype descriptions.

We formalize the phenotype "cell cycle progression: arrested" using the PATO term Arrested (PATO:0000297) and the GO process class Cell cycle process (GO:0022402):

phenotype-of some (has-part some

 GO:0022402 and has-quality some

 PATO:0000297)

We formalize the remaining phenotype description of S000029075 in a similar way and combine the individual phenotype classes using class intersection.

Phenotype descriptions based on a triple consist of an entity, a qualifier and a second entity that is used to define the respective phenotype class. For example, S000000649 is annotated with *Ionic stress resistance*: *decreased *and the additional class *Sodium chloride *(CHEBI:26710). The intended meaning of this phenotype description is that the resistance of the yeast cell to respond to sodium chloride is decreased within the specific experiment that was performed. To formalize this phenotype, we combine the PATO class *Sensitivity of a process *(PATO:0001457), the GO class *Response to chemical stimulus *(GO:0042221) and the ChEBI class *Sodium chloride *(CHEBI:26710):

phenotype-of some (has-part some

 GO:0042221 and has-quality some

 (PATO:0001457 and towards some

 CHEBI:26710))

### Using phenotypes to reveal gene functions

Our hypothesis is that phenotypes can be utilised to reveal the function of genes. For example, when a gene is knocked out with a resulting developmental phenotypic manifestation we can assume that the gene plays some role in the development of the organism. In order to validate our hypothesis and the applicability of our approach, we tested it against our ability to reproduce known gene functions for the set of yeast genes that we can recover phenotype data from SGD. We extracted the GO terms from the phenotype annotations and compared them against the GO annotation that SGD has associated with the corresponding genes. We were able to recover 11% of the GO processes annotations, 15% of the cell components annotations and 18% of the GO functions annotations found in SGD. The GO annotations we infer from the phenotypes, that are not available for the SGD, present novel candidates annotations for these gene products. For example, based on the curated single mutant phenotypes associated with CLN3 that can be found in SGD, we were able to propose the gene's involvement in the regulation of the duration of G1 phase of mitotic cell cycle. Given that *G1 cyclin *is involved in cell cycle progression and activates *Cdc28p *kinase to promote the G1 to S phase transition [31] our predicted association of the GO term *G1 phase of mitotic cell cycle *(GO:0000080) presents a novel possible GO annotation for it.

### Future research

Many of the definitions we propose do not make full use of established phenotype definition patterns that enable interoperability with ontologies of functions and processes [29,32]. However, our prime motivation in defining yeast phenotypes was to enable cross-species phenotype integration and comparison using the PhenomeBLAST and PhenomeNET methods. We have formally integrated the APO and the definitions of the APO that we created with the ontology underlying PhenomeBLAST (the software and ontology are available from http://phenomeblast.googlecode.com), and we can represent yeast phenotypes using the phenotype ontologies that were created for other species. For example, the phenotypes of S000029048 (annotated with the single phenotype *Autophagy: absent*) expressed using the Mammalian Phenotype Ontology (MP) are *Abnormal metabolism, Homeostasis/metabolism phenotype and Mammalian phenotype*. Using the Worm Phenotype Ontology (WPO), which targets an organism that is more similar to yeast than mammals, we obtain as phenotypes abnormalities of *Autophagy, Intracellular transport, Small molecule transport and Cellular processes*.

In the future we intend to evaluate this work via utilizing our ability to integrate yeast phenotypes with phenotype information from other species so as to identify interacting proteins, orthologous genes and other evolutionary or biological meaningful relations.

## Conclusion

In the future, we intend to evaluate the potential of yeast phenotype annotations to predict orthologous genes and genes involved in metabolic diseases based on comparisons of phenotypes. Furthermore, as cross-species phenotype integration progresses, we intend to update the definitions to accurately reflect more complex relations.

In the post-genomic era, the analysis and integration of phenotype data have been demonstrated as useful tools assigning genotype to phenotype correlations, providing insights in the nature of human disease and ultimately discovering novel therapeutic approaches. The challenge now remains to provide mechanisms and methods that allow such integration and analysis on a large scale that takes into account the vast amount of phenotypic information collected around the world for various species in a single framework. One such framework has been proposed based on the use of PATO and a variety of external ontologies [33] and has been successfully demonstrated to work for achieving such integration [15-18].

Here we demonstrated how yeast phenotype information could be defined based on this framework and we have successfully included yeast phenotype data in a cross species phenotype data network. As such yeast phenotype data can be integrate and manalysed with data from other species and increases their potential for discovering new genotype to phenotype correlations.

## Competing interests

The authors declare that they have no competing interests.

## Authors' contributions

GVG conceived of and created the formal phenotype definitions. RH assisted with the evaluation of the definitions and the implementation as well as extracted gene functions from phenotype annotations. Both authors wrote, have read and approved the final version of the manuscript.
